# Relationship between functional independence and psychosocial quality of life of stroke survivors undergoing outpatient rehabilitation in Maiduguri, Nigeria

**DOI:** 10.1186/s41687-019-0107-4

**Published:** 2019-03-18

**Authors:** Grace Vincent-Onabajo, Nyangwae Daniel Shaphant

**Affiliations:** 1 Independent Consultant, Ibadan, Nigeria; 2 Department of Medical Rehabilitation (Physiotherapy), College of Medical Sciences, University of Maiduguri, Bama Road, Maiduguri, Nigeria

**Keywords:** Functional independence, Health-related quality of life, Nigeria, Psychosocial quality of life, Stroke

## Abstract

**Background:**

There are divergent findings on the impact of functional independence on psychosocial quality of life (QoL) of stroke survivors.

**Aim:**

To investigate the relationship between functional independence and psychosocial QoL among stroke survivors in Maiduguri, Nigeria.

**Methods:**

A cross-sectional design was utilized, and functional independence and psychosocial QoL of consecutive stroke survivors undergoing rehabilitation were assessed with the motor subscale of the Functional Independence Measure (motor- FIM) and the psychosocial subscale of the Stroke-Specific Quality of Life Scale-12 (SS-QoL- 12) respectively. Relationships between the two variables were explored with Pearson’s correlation coefficients and multivariable regression analysis.

**Results:**

Fifty-nine stroke survivors participated in the study with a male majority (54.2%). Correlation between motor-FIM and the psychosocial subscale scores was not statistically significant (r = 0.24; *p* = 0.07) while correlation between motor-FIM and each item of the psychosocial subscale showed that a statistically significant correlation (r = 0.29; *p* = 0.02) was present for the ‘mood’ item only. Functional independence however did not statistically contribute to the multivariable regression model (R2 = 0.22; *P* < 0.01) for the ‘mood’ item after controlling for the effect of the participants’ age and sex.

**Conclusion:**

Functional independence had no independent or statistically significant relationship with overall psychosocial QoL. Further studies are therefore needed to explore modifiable factors that influence psychosocial QoL of stroke survivors in our setting.

## Introduction

Psychosocial quality of life, an important component of health-related quality of life (HRQoL), represents an individual’s perception of their psychological and social status in relation to their health conditions. In stroke, accompanying disability in terms of impairments of body functions, activity limitations and participation restrictions have been found to negatively affect overall HRQoL of survivors [1–3]. It is worthy of note that functional independence also commonly referred to as independence in activities of daily living in stroke literature has been found to be an almost consistent determinant of overall HRQoL in studies across different regions and countries [1–4].

While the impact of functional independence on overall HRQoL and physical QoL has been unanimously elucidated [1–5], the same cannot be said for psychosocial quality of life as divergent patterns have been reported about its relationship with functional independence in various studies. For instance in a study by Carod-Artal et al. [6] in Spain, stroke survivors with higher level of functional independence were found to have significantly poorer psychosocial QoL. Conversely, a study conducted in India showed that functional independence had a significant positive impact on all domains of QoL assessed including psychological and social domains [7]. Another study in Japan however found no significant relationship between functional independence and psychological domain of QoL [5].

With the largely diverse findings on the impact of functional independence on psychosocial QoL after stroke, there is a need for more information on the subject especially through setting-specific studies. This is partly because QoL entails an individual’s perception of their position in life in the context of their culture and value system. Therefore, the impact of functional independence on psychosocial QoL can be better elucidated through country-, region- or culture-specific studies.

In Nigeria, as in other African countries, the incidence and prevalence of stroke are on the increase [8, 9]. In recent times, a deadly terrorism and insurgency sprung up in some states and a specific geopolitical region of Nigeria with significant impact on the lives of the general population. With the peculiarity of the experience of residing in the affected areas, and the appropriateness of analysing and understanding health-related QoL in a setting- or culture-specific context, the authors sought to identify the relationship between functional independence and psychosocial QoL among stroke survivors in Maiduguri, the epicenter of the deadly insurgency and terrorism in Nigeria. With psychosocial QoL operationalised in terms of six items of the Stroke-Specific Quality of Life scale-12 namely thinking, family roles, social roles, personality, mood and energy, the primary aim of this study was to identify the relationship between functional independence and global, and specific components of psychosocial QoL of stroke survivors in Maiduguri, Nigeria. The status of the stroke survivors as it concerns the specific components of psychosocial QoL was also explored.

## Methods

### Study design

The study was a multicentre hospital-based cross-sectional survey.

### Study setting

The study was conducted at the outpatient physiotherapy facilities of two out of the three government hospitals with such facilities in Maiduguri, Borno State. Maiduguri is the capital city of Borno State, in North-East geo-political region of Nigeria with an estimated population of 1,112,400 as at 2015. Maiduguri became the hot bed of terrorist activities in 2009 and thousands of people have been killed while many businesses, schools, homes and social amenities were destroyed. In 2014 (the year the study was conducted), one of the deadliest attacks on Maiduguri occurred on March 14 [10].

The two outpatient physiotherapy facilities that constituted the study sites provide physiotherapy services for the people in the State and other neighbouring states whenever they present themselves for consultation and waiting lists are usually not utilized. Also due to the lack of standalone community, and inpatient rehabilitation centers in the State, as in many other States in Nigeria, stroke patients requiring physiotherapy rehabilitation services have very few options to select from in terms of care setting and many are left with the option of outpatient rehabilitation services while those who prefer and can afford home-based physiotherapy services are also able to avail themselves of such services [11].

### Participants

Fifty-nine community-dwelling stroke survivors were recruited during their rehabilitation appointments at the study sites. To be eligible to participate in the study, stroke survivors expressed their willingness to be involved through the completion of informed consent forms; were aged 18 years and above; and were capable of verbal and coherent communication as documented in each stroke survivor's medical records.

### Study instruments

Demographic and time since stroke onset data were obtained from the participants (or in some cases from the medical records) and recorded in data forms prepared for that purpose.

The motor subscale of the Functional Independence Measure (motor-FIM) [12] was interviewer-adminstered to assess functional independence in the domains of self-care, sphincter control, mobility, and locomotion. Level of independence is scored on a seven-point scale of 1 to 7 for the 13 items of the motor subscale. Total motor-FIM score was obtained by simple summation of the item scores, and can range from 13 to 91 with higher score depicting higher level of independence. The motor-FIM has an excellent internal consistency (Cronbach’s alpha ranges from 0.88 to 0.91) and a good concurrent validity (intraclass coefficient ranges from 0.83 to 0.87) with the Barthel Index [13].

The psychosocial QoL of the participants was assessed using the psychosocial subscale of the 12-item Stroke-Specific Quality of Life scale (SSQoL-12). The SS-QoL-12 is a short version of the original 49-item SS-QoL [14]. The psychosocial subscale has 6 items that assesses thinking, family relation, social role, personality, mood, and energy, and the items are scored on a 5-point Likert-type scale which ranges from 1 to 5. Total score on the psychosocial subscale of the SS-QoL-12 is obtained by calculating the unweighted average of the scores of the 6 items, and ranges from 1 to 5 (the higher the score, the better the QoL). The SS-QoL-12 has been shown to possess acceptable measurement properties such as validity and reliability among patients receiving rehabilitation [15]. Internal consistency (Cronbach’s alpha) for the psychosocial SS-QoL-12 ranges from 0.77 to 0.89 while the validity of the SS-QoL-12, obtained through comparison with the original version of the SS-QoL (SS-QoL-49) as the criterion measure, was good as shown by the negligible mean difference (− 0.03; 95% Confidence Interval − 0.07 – 0.01) between scores obtained on the two versions by the same group of stroke patients [15].

### Procedure

Ethical approval for the study was obtained from the Research and Ethical Committee of the University of Maiduguri Teaching Hospital, Maiduguri, Nigeria. Consecutive and consenting stroke survivors receiving physiotherapy at the time of the study and who met the eligibility criteria were invited into the study. All data were obtained from the participants through face-to-face interview while data on time since stroke onset was verified using individual stroke survivor’s medical records. All data were collected from June to July 2014 by the second author (NSP) who is a physiotherapist.

### Statistical analyses

Descriptive statistics of mean and standard deviation, percentages and frequencies were used to present the demographic, time since stroke onset, functional independence and psychosocial QoL data of the study participants.

Pearson’s correlation coefficients (bivariate analysis) [16] were calculated to assess the relationships between the motor-FIM score and each of overall and item scores of the SSQoL-12 psychosocial subscale. Multivariable linear regression [17] was conducted to determine whether functional independence significantly and independently influenced specific aspect(s) of psychosocial QoL that yielded significant correlation in the bivariate analysis (only ‘mood’ was found to significantly correlate with motor-FIM score) while controlling for the likely confounding effects of age, and gender on psychosocial QoL. Therefore a regression analysis, using the ‘enter’ method which is the default method, was conducted only for the mood item (dependent variable) which had functional independence, age, and gender as the independent variables. Level of statistical significance was set at α = 0.05 and two-tailed *p* values less than 0.05 led to rejection of the a priori null hypothesis that functional independence will have no significant impact on psychosocial QoL of stroke survivors. Statistical analyses were performed with SPSS version 15 (SPSS Inc., Chicago, IL., USA).

## Results

Of the 59 stroke survivors that participated in the study, there were more male stroke survivors (*n* = 32; 54.2%) than females (*n* = 27; 45.8%). Mean age of the participants was 53.85 (SD =17.29) years while mean time since stroke onset was 15.12 (SD = 22.01) months. Mean motor-FIM and SS-QoL-12 psychosocial subscale score was 62.80 (SD = 25.15) and 3.34 (SD = 0.89) respectively. Table 1 shows the mean scores for the items of the SSQoL-12 psychosocial subscale.

**Table 1:** Mean values obtained for the six items of the SS-QoL-12 psychosocial subscale

Item		*Mean (SD)
Thinking:	I had trouble remembering things	3.80 (1.51)
Family Relation:	I felt I was a burden to my family	3.63 (1.62)
Social Role:	My physical condition interfered with my social life	2.98 (1.68)
Personality:	My personality has changed	3.10 (1.66)
Mood:	I was discouraged about my future	3.95 (1.47)
Energy:	I was too tired to do what I wanted to do	2.66 (1.63)

*maximum score possible is 5; higher score depicts better QoL

The outcome of the correlation analysis between motor-FIM score and SS-QoL-12 psychosocial subscale score showed a weak and positive correlation that lacked statistical significance (r = 0.24; *P* = 0.07). Correlation coefficients that range from − 0.3 to + 0.3 are regarded as weak [18]. Correlation between motor-FIM score and each of the 6 items of SS-Qol-12 psychosocial QoL showed that ‘mood’ was the only item with a statistically significant correlation with motor-FIM score and the correlation was weak at r = 0.29 (Table 2).

**Table 2:** Pearson’s Correlation matrix for relationship between functional independence and overall, and components of psychosocial QoL

Variable	1	2	3	4	5	6	7	8
1 FI	1							
2 Psychosocial QoL	.24	1						
3 Thinking	−.002	.42**	1					
4 Family relation	.02	.69***	.16	1				
5 Social role	.17	.44**	−.01	.22	1			
6 Personality	.07	.62***	.06	.37**	.03	1		
7 Mood	.29*	.51***	.15	.16	.11	.20	1	
8 Energy	.20	.67***	.15	.41	.06	.37**	.22*	1

*FI* Functional independence; ***P < 0.001; **P < 0.01; *P < 0.05 Correlation coefficient (r)

Results of the regression analysis which had ‘mood’ as the dependent variable and functional independence, age and sex as the independent variables yielded R^2^ = 0.22 at *P* < 0.01 (Table 3). Functional independence however had no statistically significant contribution to the regression model (β = 0.05; *P* = 0.75) while age (β = − 0.37; *P* = 0.01) and sex (β = 0.28; *P* = 0.03) were significant contributors to the regression model. The regression coefficients (β) showed that age contributed more to the regression model than sex.

**Table 3:** Multivariable Regression Analysis for Impact of Functional Independence on Psychosocial (Mood) Quality of Life

Variable	Regression Coefficient (β)	*P* value	95% CI
FI	0.05	0.75	−0.01 to −0.02
Age	−0.37	0.01*	−0.06 to −0.01
Sex	−0.28	0.03*	− 1.54 to − 0.07
R2 = 0. 22		0.01*	

CI: Confidence Interval

*statistically significant at *P* < 0.05

## Discussion

The centrality of functional independence as an outcome after stroke and its consistent and significant effect on almost all other outcomes such as health care utilization, caregivers’ burden and global QoL dictate that the effect of functional independence on an important outcome such as psychological QoL be explored in our environment. This is more so as existing studies have reported largely contradictory findings on the relationship between functional independence and psychosocial QoL [5–7]. The findings of the present study correspond with that of Mutai et al. [5] which showed a lack of significant relationship between functional independence and the psychological component of QoL of stroke survivors in Japan. However, a study conducted in Spain showed that highly functionally independent stroke survivors experienced deterioration in their psychosocial quality of life [6]. One of the reasons adduced for the trend was the likely dissatisfaction of high functioning stroke survivors with their inability to fully return to pre-stroke levels of functional independence with attendant negative impact on their psychosocial QoL. Another study of stroke survivors in India identified functional dependence as an independent predictor of diminished social QoL [7] and the inability to actively engage in social activities due to functional limitation could be responsible for the finding. The present study however also explored the relationship between functional independence and individual items that constitute psychosocial QoL as assessed by the SS-QoL-12 and found a significant relationship with the ‘mood’ item in the bivariate but not the multivariate analysis.

The ‘mood’ item of the SS-QoL-12 psychosocial subscale that was found to be statistically and positively related with functional independence has the statement “I was discouraged about my future”. Since higher scores on the two instruments used to assess the functional independence and psychosocial QoL represent a positive outcome, the implication of this finding is that stroke survivors who were functionally independent had better perception of their future and vice versa. It could therefore be inferred that being functionally independent may provide stroke survivors with a positive outlook about the future. However the outcome of the bivariate analysis cannot rule out the bidirectionality of the relationship between the ‘mood’ item and functional independence. This implies that while functional independence affects mood, mood can also influence the functional independence status of stroke survivors. The fact that several studies have been able to identify the significant influence of psychological variables such as depression [19, 20], and apathy [19] on functional recovery after stroke evidences the bidirectional relationship between the two main constructs.

It is important to note that the highest mean score was observed in the ‘mood’ item which is indicative of comparatively better perception by the stroke survivors in the study of the future as assessed by the item while the lowest mean score was recorded for ‘energy’ item. This finding on relatively better mood may suggest that the stroke survivors stayed upbeat not only in the face of their health challenges but also in spite of the environmental challenges in their domiciles which was largely characterized by a prolonged insurgency and attendant socio-economic woes. Religion, and the belief systems of Nigerians have consistently been implicated as sources of optimism, and coping mechanism in the face of challenging life situations [21, 22]. It is also important to note that, although not statistically significant, correlations between functional independence and four out of the six items of the psychosocial domain of the SS-QoL-12 were positive which imply that the more functionally independent stroke survivors had better psychosocial QoL. However, an unexpected and difficult to explain negative correlation was observed between functional independence and the ‘thinking’ component of psycholosocial QoL with implication that functionally independent stroke survivors had more trouble remembering things compared to those who were dependent. It is equally important to note that the statistically significant positive correlation between ‘mood’ and functional independence obtained in the bivariate analysis was not observed in the multivariate analysis. Rather, it was age and sex that were found to significantly and independently influence psychosocial QoL. It is worthy of mention that comparisons of these findings could not be made as there appears to be no existing literature that explored the impact of functional independence on individual components or items of psychosocial QoL.

While post-stroke rehabilitation, especially physiotherapy emphasizes the restoration of functional independence, finding a lack of significant association between functional independence and psychosocial QoL in this study is an indication of the need for further studies on the determinants of stroke survivors’ psychosocial QoL in our environment. Simply put, an implication of the finding of the study is that a sole focus on improving functional independence through appropriate rehabilitation strategies may not translate to a positive perception of psychosocial status or better psychosocial QoL among stroke survivors. In fact, the earlier mentioned study by Carod-Artal et al. [6] found that stroke survivors with better functional independence reported poorer psychosocial QoL. Hence, identification of factors that can positively affect psychosocial QoL of stroke survivors is of utmost importance. This is especially necessary when the negative impact of poor psychosocial status after stroke is considered. For instance, emotional QoL during inpatient rehabilitation was reported to predict global HRQoL two and half years after [23]. Also, there appears to be a significant relationship between psychosocial QoL and suicide ideation and attempt [24, 25]. It can therefore be expected that with proper identification and documentation of factors that influence psychosocial QoL, effective interventions and strategies capable of enhancing stroke survivors’ psychosocial QoL can be devised. This identification can be achieved through well designed prospective cohort studies. Clinical trials of community-based culturally sensitive interventions to improve psychosocial QoL would also be required. Additionally, rehabilitation professionals and policy makers would need to specifically assess and pointedly address psychosocial QoL among their stroke patients and not utilize functional independence status as a proxy of QoL post-stroke.

### Limitations of the study

The small size of the sample and the use of a non randomised sampling technique limit the representativeness of the study sample. Similarly, the study’s hospital-based design limits the generalisability of findings. Reverse causality in the relationship between functional independence and the ‘mood’ component of psychosocial QoL could not be ruled out given the cross-sectional design of the study, hence the need for future prospective studies. Furthermore, the potentially bidirectional nature of the relationship between functional independence and mood requires cautious interpretation of findings of the study.

## Conclusion

Functional independence had no statistically significant relationship with psychosocial QoL of the stroke survivors while a significant relationship was observed with the ‘mood’ component of psychosocial QoL. However, the fact that functional independence had no significant and independent influence on psychosocial QoL either as a global construct or in terms of its components implies that achieving functional independence after stroke may not necessarily translate to the enhancement of an important post-stroke outcome such as psychosocial QoL. Therefore, for effective enhancement of psychosocial QoL of stroke survivors in Nigeria, further studies are required to identify its determinants and predictors.

## Abbreviations

HRQoL: Health-Related Quality of Life
Motor-FIM: Motor subscale of the Functional Independence Measure
QoL: Quality of Life
SD: Standard Deviation
SS-QoL: Stroke-Specific Quality of Life
SS-QoL-12: 12-item Stroke-Specific Quality of Life scale
SS-QoL-49: Original version of the Stroke-Specific Quality of Life
